# Quantitative assessment of myocardial mechanics in patients with cardiac amyloid using cardiovascular magnetic resonance myocardial feature tracking

**DOI:** 10.1186/1532-429X-17-S1-Q28

**Published:** 2015-02-03

**Authors:** Shazia T Hussain, Sebastian Buss, Shelby Kutty, Henning Steen, Dirk Lossnitzer, Philipp B Beerbaum, Pablo Lamata, Andreas Schuster

**Affiliations:** 1 Imaging Sciences, Kings College Hospital, London, UK; 2 Cardiology, Papworth Hospital, Cambridgeshire, UK; 3 Cardiology, University of Heidelberg, Heidelberg, Germany; 4 University of Nebraska, Omaha, NE; 5 Department of paediatric cardiology, Hannover Medical School, Hannover, Germany; 6 Cardiology and Pneumology, Georg-August-University, Gottingen, Germany

## Background

Cardiovascular magnetic resonance (CMR) feature-tracking (FT) allows the quantitative assessment of complex ventricular mechanics such as strain, twist and untwist. LV twist results from the dynamic interplay between systolic clockwise rotation of the base and a counterclockwise rotation of the apex followed by untwisting during diastole.

We sought to determine whether strain, myocardial twist and untwist rates could be measured by CMR-FT and hypothesized that twist and untwist rates would be reduced in patients with amyloid disease as a consequence of systolic and diastolic dysfunction.

## Methods

The CMR images of 62 patients with biopsy-proven amyloid, and 10 healthy volunteers were assessed with CMR-FT post-processing software (TomTec, Germany). All subjects had routine steady state free precession (SSFP) cine imaging in the short axis and 4-chamber orientations at 1.5 Tesla. Peak longitudinal (Ell), radial (Err) and circumferential endocardial (Ecc_endo_) and circumferential epicardial (Ecc_epi_) strain was measured. Additionally, the rotation of the basal and apical slices was measured and global LV twist θ was calculated as the difference between the overall counterclockwise (positive) rotation at the apex (φ_apex_) and the overall clockwise rotation at the base (viewed from apex), θ = φ_apex_- φ_base_.

**Figure 1:**
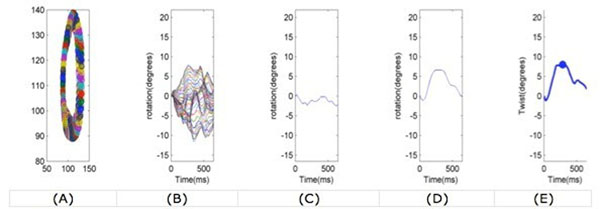
(A) Example of the temporal evolution of the myocardial contours in 2D. (B) Temporal evolution of the angular rotation of the 48 control points of the contour. (C) Average rotation from the 48 control points in the basal slice. (D) Average rotation in the apical slice. (E) Global LV twist.

Peak twist and untwist rates were calculated using MATLAB software (figure 1).

## Results

**Figure 2:**
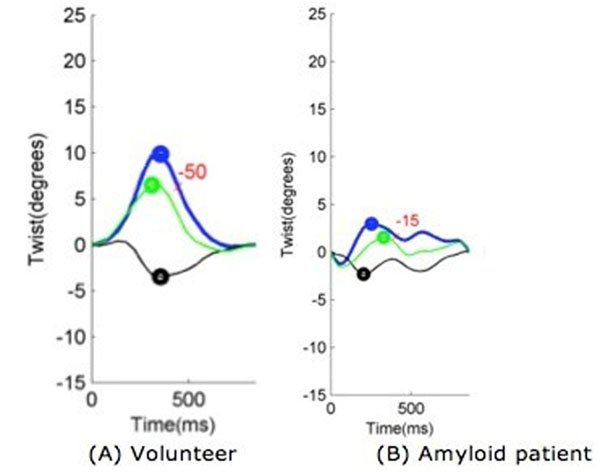
**Overall twist.** Overall twist (purple line) is calculated from the difference between counterclockwise apical rotation (green line) and the clockwise basal rotation (black line). The untwist rate is marked in red. The results in the amyloid pt (B) are reduced compared to those of the volunteer (A)

We observed a significant difference in myocardial twist and the untwist rate between the patients and the volunteers (p<0.05, table 1 and figure 2). Whilst there was a significant reduction in longitudinal and circumferential strain in the amyloid cohort compared to the volunteers (p<0.05, table 1) the radial strain remained unchanged (p>0.05, table 1).

**Table 1:** Mean values of peak strain, peak twist and untwist rates in the amyloid cohort and in volunteers.

Strain/Twist	Group	Mean value - peak % (+/-SD)	Sig (2 tailed)*
Ell	Volunteer	-18.78 (+/-3.7)	p<0.005
		
	Amyloid	-6.73 (+/-11)	

Err	Volunteer	36.97 (+/-26.5)	p=0.510
		
	Amyloid	35.00 (+/-35)	

Ecc endo	Volunteer	-25.96 (+/-11.6)	p<0.005
		
	Amyloid	-17.81 (+/-11.4)	

Ecc epi	Volunteer	-17.26 (+/-9.6)	p<0.005
		
	Amyloid	-10.34 (+/-9.3)	

Twist(degrees)	Volunteer	8.05 (+/-6.1)	p=0.025
		
	Amyloid	3.05 (+/-6.42)	

Untwist-rate (degrees/s)	Volunteer	-65.5 (+/-46.42)	P=0.04
		
	Amyloid	-19.2 (+/-45.6)	

* Significance tested with an unpaired t-test

## Conclusions

This study demonstrates the feasibility of measuring complex mechanics from routine cine images in patients with amyloid using CMR-FT.

The reduction in strain and twist in the amyloid patients is likely to reflect systolic dysfunction. In addition reduced untwist rates may be a measure of diastolic dysfunction.

Larger scale studies are required to validate this further, however, we have demonstrated that CMR-FT has the potential to define diastolic dysfunction from routinely acquired CMR cine images.

## Funding

N/A.

